# An Unusual Case Report of Duodenojejunal Flexure Tumor With a Systematic Review of the Literature

**DOI:** 10.7759/cureus.76196

**Published:** 2024-12-22

**Authors:** Arushi Choudhary, Shubhransu Patro, Vedavyas Mohapatra, Swati Das, Akruti Mishra, Purusottam Misra, Sham Charan Kossuru, Preetam Nath, Suprabhat Giri

**Affiliations:** 1 General Medicine, Kalinga Institute of Medical Sciences, Bhubaneswar, IND; 2 Surgical Gastroenterology, Kalinga Institute of Medical Sciences, Bhubaneswar, IND; 3 Radiodiagnosis, Kalinga Institute of Medical Sciences, Bhubaneswar, IND; 4 Pathology, Kalinga Institute of Medical Sciences, Bhubaneswar, IND; 5 Gastroenterology and Hepatology, Kalinga Institute of Medical Sciences, Bhubaneswar, IND

**Keywords:** adenocarcinoma, duodenojejunal flexure tumor, enteroenteric fistula, gastrointestinal stromal tumor, small intestinal tumor

## Abstract

The small intestine is the longest segment of the gastrointestinal (GI) tract, but cancers in the small intestine are infrequent. The duodenojejunal (DJ) flexure is an uncommon site for tumors, and those located in these sites are difficult to identify and manage properly. Their rarity, along with ambiguous symptoms that can be readily misattributed to milder conditions, results in a delayed diagnosis when the tumors have significantly advanced. We described the case report of a middle-aged woman presenting with features of intestinal obstruction with gastrointestinal bleeding, showing enteroenteric fistula on enteroscopy and cross-sectional imaging, which was later diagnosed as a case of adenocarcinoma treated with surgical resection. We also systematically reviewed the current literature on DJ flexure tumors and compiled data based on various clinical presentations, radiological findings, associated syndromes, management, and outcomes.

## Introduction

Despite the small intestine constituting over 75% of the length and 90% of the mucosal surface area of the gastrointestinal (GI) tract, small intestine cancer is relatively rare, accounting for only 1% of gastrointestinal malignancies [1]. Small bowel tumors encompass around 40 different histological subtypes. Benign varieties of small intestinal tumors include hemangiomas, lipomas, and hamartomas (associated with Peutz-Jeghers syndrome), while malignant tumors encompass adenocarcinomas, gastrointestinal stromal tumors (GISTs), neuroendocrine tumors (NET), and lymphomas. In India, the incidence of small intestinal cancers is extremely rare, with lymphomas being the most common type of malignancy within this group [2,3].

One of the sites in the small bowel is the duodenojejunal (DJ) flexure. Tumors located in these are challenging to diagnose and treat effectively. Their infrequency, combined with vague symptoms (vomiting, weight loss, abdominal distension, melena, and pallor), which may be easily misattributed to less severe ailments, leads to delayed diagnosis when the tumors have progressed considerably. The incidence of small bowel tumors has been rising in recent years, with India having the highest annual percentage change in incidence among men. In 2020, the age-standardized incidence rate of small intestinal cancer in the world and India was 0.6 and 0.3 per 100,000, respectively [4]. This increase can be attributed to advancements in diagnostic techniques and modalities. Advancements in imaging technology, endoscopic techniques, and other diagnostic instruments that have improved the capacity to identify these cancers sooner and with greater precision account for the noted increase in incidence rates [2,4].

Accurate and timely diagnosis is crucial for improving patient outcomes, but the subtle nature of these initial symptoms poses a significant hurdle. When subtle clues are missed, the disease usually presents at an advanced stage. At our center, we came across a case diagnosed with DJ flexure adenocarcinoma with enteroenteric fistula between the D3 segment of the duodenum and jejunum and growth at the descending colon. Initially, before histopathological diagnosis, the differential diagnosis of tuberculosis and Crohn's disease of the small intestine was also kept in mind. This prompted us to review all the reported DJ flexure tumors systematically. We have compiled data based on various clinical presentations, associated syndromes, the diagnostic modality used, and the management of each.

## Case presentation

A 35-year-old woman presented with vomiting and abdominal pain for four days. She had two episodes of coffee-ground vomitus, containing food particles with no specific triggers for the symptoms. The abdominal pain was present in the left lower quadrant for the past six months, with a gradual onset, intermittent nature, usually occurring five days before her menstrual cycle, and the pain was relieved with medication. The patient also complained of loss of appetite for the past six months. There was no history of fever, weight loss, cough, loose stools, blood in stools, or black stools. The patient has been a known case of hypothyroidism for the past eight years and was previously admitted in June 2023 for generalized weakness. She was diagnosed with severe anemia and occult gastrointestinal (GI) bleeding and received iron injections. Upper GI endoscopy and colonoscopy at that time showed no abnormalities.

**Figure 1:**
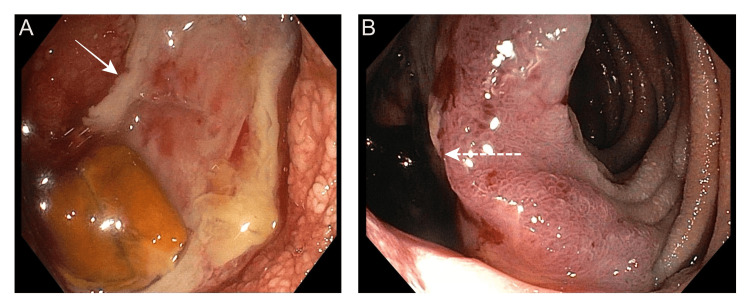
Push enteroscopy image showing (A) an ulcerated area (arrow) with significant luminal narrowing at the duodenojejunal flexure and (B) a fistulous tract (dotted arrow) proximal to the site of narrowing.

Clinical examination showed no findings except for the presence of pallor. The patient's hemoglobin (Hb) level was 4.4 g/dL, with an iron profile showing severe iron deficiency (serum iron=10 mcg/dL). The patient received three units of packed red blood cells and intravenous iron supplementation (2 g ferric carboxymaltose). Ultrasound of the abdomen and pelvis showed thickened and edematous jejunal bowel loops and mildly bulky ovaries with multiple follicles. A contrast computed tomography (CT) of the abdomen showed a segment of the proximal jejunal loop with edematous wall thickening and possible adhesion of adjacent jejunal loops, suggesting an infective/inflammatory etiology with bilateral mildly bulky ovaries with multiple follicles, possibly indicative of polycystic ovarian disease. A push enteroscopy was performed, and it showed an ulcerated area with significant luminal narrowing at the DJ flexure and a fistulous tract proximal to it (Figure 1).

**Figure 2:**
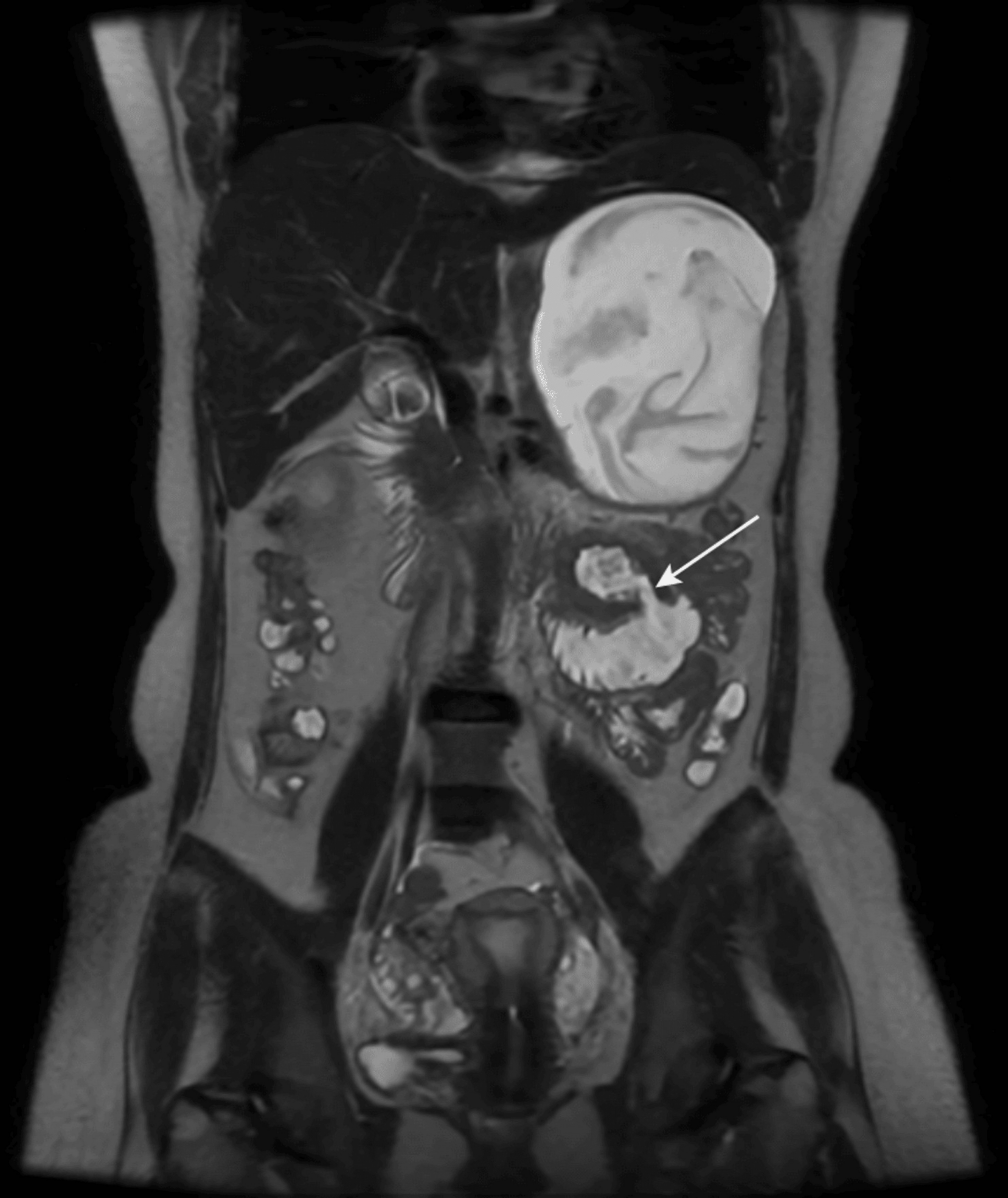
Magnetic resonance enterography showing irregular heterogeneously enhancing wall thickening showing diffusion restriction seen involving the distal duodenum proximal jejunum and duodenojejunal flexure with maximum wall thickness ~11.2 mm over a length of ~4.7 cm with surrounding diffuse inflammatory changes and abnormal communication (arrow) between the duodenojejunal flexure and jejunal lumen, likely enteroenteric fistula.

**Figure 3:**
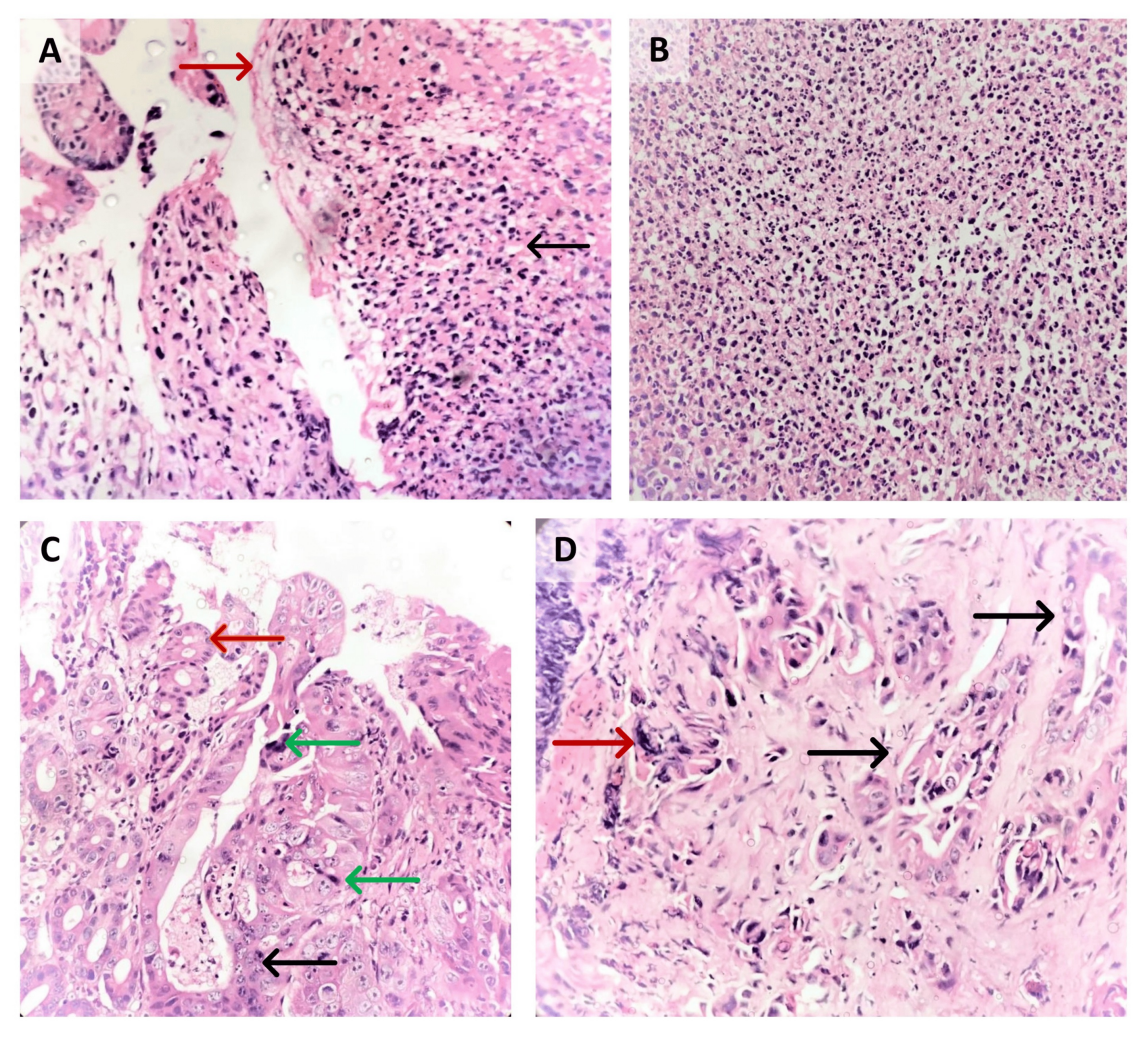
(A) Hematoxylin and eosin (H&E) (400×): small intestinal tissue from the ulcer site showing surface ulceration (red arrow) with the formation of granulation tissue (black arrow) and (B) moderate mixed inflammatory cell infiltration in the lamina propria. (C) Bits from the fistula site showing normal intestinal mucosa (red arrow) with adjacent area showing an infiltrative tumor with tumor cells arranged in glandular pattern (black arrow) and atypical mitotic figures (green arrow) and (D) tumor cells arranged in glandular pattern (black arrow) with cells being highly pleomorphic with high nuclear/cytoplasmic ratio, scant cytoplasm, vesicular nucleus with prominent nucleoli, and atypical mitotic figures (red arrow).

Six bits of biopsies were taken, and the biopsy showed ulcerative jejunitis in five bits and suspected malignant cells in one bit. The patient was readmitted with the complaint of melena and vomiting for 5-6 days. A magnetic resonance (MR) enterography was done, which revealed irregular heterogeneously enhancing wall thickening with diffusion restriction involving the distal duodenum, proximal jejunum, and DJ flexure with an abnormal communication between the D3 segment of the duodenum and jejunal lumen, likely indicating an enteroenteric fistula (Figure 2). A repeat push enteroscopy with biopsy was performed, showing features suggestive of infiltrating adenocarcinoma (Figure 3).

The patient underwent diagnostic laparoscopy, and intraoperatively, a growth of the DJ flexure infiltrating the proximal jejunum, descending colon, and inferior mesenteric artery was found. The surgical excision of the DJ flexure with proximal jejunum and descending colon with D3 to jejunum anastomosis with Hartmann's procedure with feeding jejunostomy (FJ) and end descending colostomy was done. The patient recovered and was discharged in stable condition on a full oral diet with FJ in situ and on chemotherapy by folinic acid, fluorouracil, and oxaliplatin (FOLFOX) and bevacizumab.

## Discussion

Systematic review

The present systematic review was conducted per the updated Preferred Reporting Items for Systematic Reviews and Meta-Analyses (PRISMA) guidelines [5].

Database Search and Study Inclusion

Electronic databases of Medical Literature Analysis and Retrieval System Online (MEDLINE), Embase, and ScienceDirect were searched from inception to July 2024 for the titles and abstracts using the following keywords: (Duodenojejunal OR DJ Flexure) AND (Tumor OR Mass OR Cancer). There were no restrictions on language provided that the study outcomes were mentioned in the article. Two independent reviewers evaluated the titles and abstracts of the retrieved papers and examined the complete texts for eligibility before inclusion. The bibliography of the listed studies was examined for relevant studies. A third reviewer adjudicated any discrepancies. Studies included in this review were case reports and case series reporting the etiology and management of DJ flexure tumors. Review articles, correspondences, conference abstracts, and editorials were excluded. Studies without relevant clinical data or incomplete data were also excluded.

Data Extraction and Quality Assessment

Data were collected in a structured extraction form by two reviewers. The record contained the following parameters of each study: title, first author, year of publication, country, number of patients, age and gender, clinical presentation, radiological findings, surgical details, tumor etiology, and long-term outcomes. Two independent reviewers assessed the quality of the included studies as per the Joanna Briggs Institute critical appraisal checklist for case reports [6]. A third independent individual was consulted in case of any discrepancy.

Characteristics of the Included Studies

**Figure 4:**
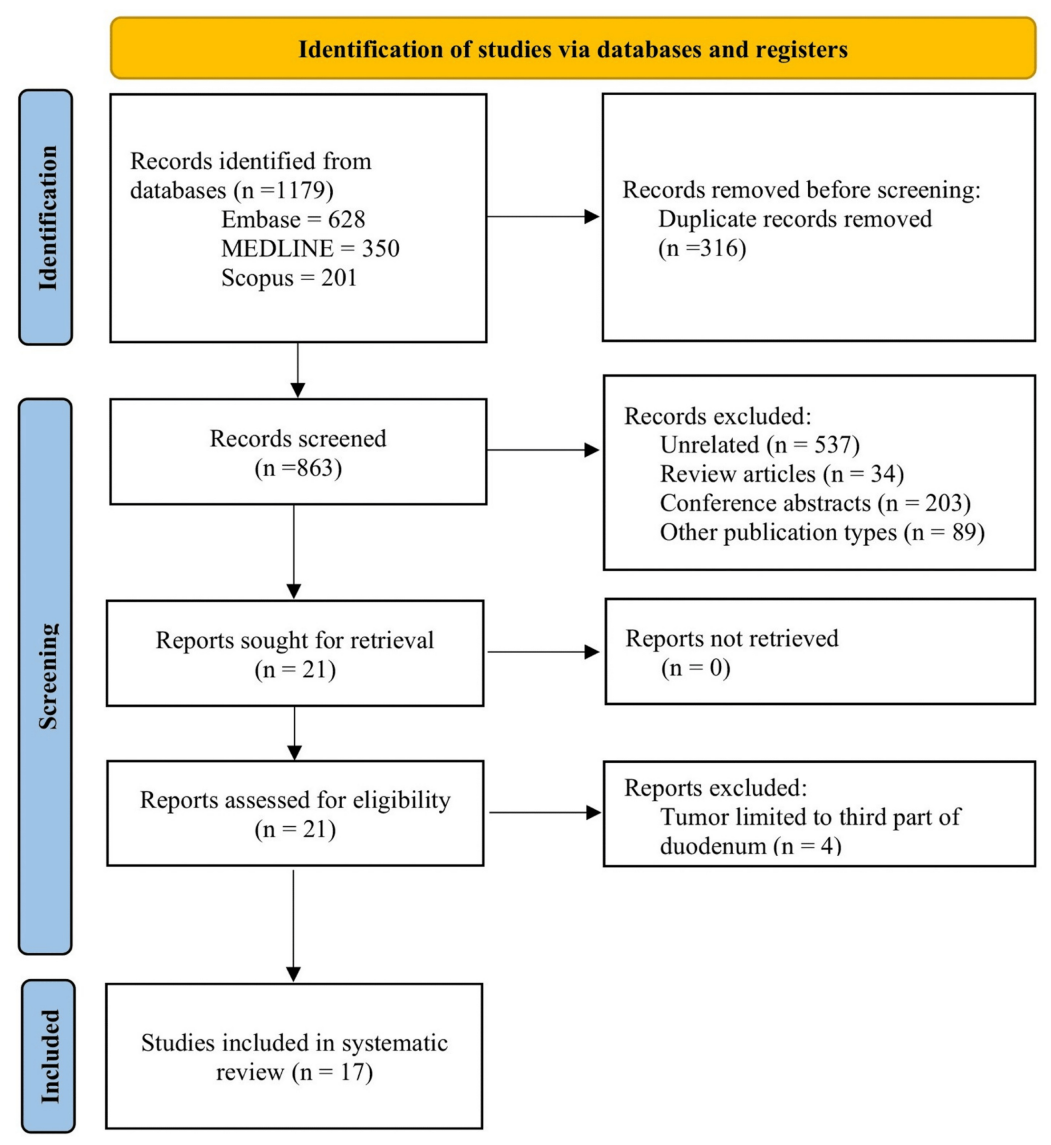
PRISMA flowchart showing the study identification, selection, and inclusion process. PRISMA, Preferred Reporting Items for Systematic Reviews and Meta-Analyses; MEDLINE, Medical Literature Analysis and Retrieval System Online

A total of 1,179 records were identified with the search strategy, of which 17 case reports/series were included in the final analysis [3,7-22]. Figure 4 shows the PRISMA flowchart for the study selection and inclusion process.

Table 1 shows the demographic, clinical, radiological, surgical, and pathological details, as well as the long-term outcomes and the quality analysis of the study. There were 14 case reports and three case series. The majority of the reports were from Europe, followed by Asia.

**Table 1:** Baseline characteristics and outcome of the studies included in the present review CT, computed tomography; DJ, duodenojejunal; GIST, gastrointestinal stromal tumor; HPF, high-power field; N/A; not available; Hb, hemoglobin

Author, year, and country	Number of patients	Age and gender	Presentation	Imaging	Surgery	Diagnosis	Associated diseases	Other management	Long-term outcome	Quality
MacGowan et al., 1996, Ireland [7]	1	59/female	Vomiting, postprandial fullness, and weight loss	Barium meal and follow-through	Whipple's resection	Adenocarcinoma	Celiac disease	Gluten-free diet	Recurrence after five years, death due to obstruction and perforation	8
Lutterer et al., 1999, Germany [10]	1	64/female	Melena	Barium follow-through showed a tumor at the DJ flexure	Resection and anastomosis	Leiomyoma	-	-	-	5
Pisanu et al., 2001, Italy [11]	1	69/female	Asthenia	CT showed a 5.5 cm mass in Treitz's angle	Resection and anastomosis	GIST	Associated gastric GIST	None	No recurrence or metastasis within 47 months	8
Cienfuegos et al., 2009, Spain [12]	1	38/male	Pain in the abdomen and vomiting	CT showed jejunojejunal intussusception with a solid mass at the DJ flexure	Pancreaticoduodenectomy with simultaneous resection of 23 cm of the jejunum	Well-differentiated adenocarcinoma	Peutz-Jeghers syndrome	-	No recurrence within 11 years of follow-up	8
Chen et al., 2012, China [13]	1	42/male	Abdominal pain and vomiting for 14 days	CT showed duodenojejunal intussusception with a large, lobulated, intraluminal mass	Resection and anastomosis	Poorly differentiated neuroendocrine carcinoma	Peutz-Jeghers syndrome	None	Local recurrence and hepatic metastasis after four months and died one month after	8
Jerraya et al., 2013, Tunisia [14]	2	72/male and 52/male	Case 1, anemia; case 2, abdominal pain	CT showed tumors in both cases	Resection and end-to-end anastomosis	Leiomyosarcoma	-	-	Case 1, N/A; case 2, died after three years due to disease recurrence	6
Manxhuka-Kerliu et al., 2014, Kosovo [15]	1	30/female	Pain in the abdomen and vomiting	CT showing an infiltrating mass of 10 cm at the DJ flexure	Resection and end-to-end anastomosis	Malignant GIST	None	None	-	7
Prabhu et al., 2014, India [3]	1	50/female	Pain in the abdomen, vomiting, and weight loss	CT showing stricturous lesion in the proximal part of the jejunum	Resection and end-to-side anastomosis	Moderately differentiated adenocarcinoma	None	Adjuvant chemotherapy	-	7
Tanaka et al., 2015, Japan [16]	8	51.3±11.7; male, 5; female, 3	-	-	Laparoscopic resection	GIST, G1 (≤5/50 HPF): 7; G2 (>5/50 HPF): 1	-	-	Recurrence: 0	4
	11	56.6±13.0; male, 7; female, 4	-	-	Open resection	GIST, G1: 10; G2: 1	-	-	Recurrence: 1	
Kumar et al., 2015, India [8]	6	35-70; 4, male; 2, female	-	-	Resection and anastomosis	Moderately differentiated adenocarcinoma, 3; GIST, 2; pancreatic choristoma, 1	Chronic pancreatitis in the case of choristoma	-	-	4
Caruso et al., 2016, Italy [17]	1	73/male	Melena and anemia	CT showed an 8 cm vascular mass at the angle of Treitz	Resection and end-to-side anastomosis	Malignant GIST	None	Adjuvant imatinib	No recurrence at four months	8
Ines et al., 2016, Tunisia [18]	1	40/male	Epigastric pain and vomiting	CT showed a 6 cm thickened wall with enhancement at the DJ flexure	Resection and end-to-end anastomosis	Well-differentiated adenocarcinoma	Celiac disease for 35 years	None	No recurrence at six months	8
Ludmir et al., 2016, USA [19]	1	72/female	Nausea, vomiting, and abdominal pain	CT showed a thickened bowel wall at the DJ flexure with partial calcification	Resection and anastomosis	Mixed adenoneuroendocrine carcinoma	None	-	-	6
Fujimoto and Osada, 2019, Japan [20]	1	91/female	Anemia and vomiting	Duodenojejunal intussusception	Resection and end-to-end anastomosis	Malignant GIST	-	-	Normal Hb at six months	6
Lee et al., 2020, South Korea [21]	1	56/female	Melena	CT showed a well-defined exophytic mass measuring 40 mm×45 mm at the DJ flexure	Resection and side-to-side anastomosis	High-grade GIST	-	-	-	5
Köroğlu et al., 2022, Turkey [22]	1	56/male	-	Heterogeneously enhanced mass at the DJ flexure with ^68^Ga-DOTATATE uptake	Resection and anastomosis	Low-grade GIST	-	-	-	4
Mukherjee et al., 2023, India [9]	1	15/female	Pain in the abdomen and vomiting	CT showed concentric heterogenous luminal narrowing near the DJ flexure	Resection and side-to-side anastomosis	Poorly differentiated adenocarcinoma	None	Adjuvant chemotherapy	Normal at three months of follow-up	8

Discussion

Small intestinal cancers are extremely rare, with adenocarcinoma and lymphoma being the most common types of malignancies within this group. The average age at which small intestinal tumors present is 59.5 years, contrary to our case of a 35-year-old woman. Out of the 41 reported cases of DJ flexure tumors worldwide, the mean age was found to be 54.2 years; 23 (56%) were men, and 17 (41%) were women. Currently, no clear gender preference has been identified. However, data suggest that men may be more prone to developing malignant forms of these tumors, contrary to our case report. Approximately 40% of small bowel tumors are adenocarcinomas, with nearly half of these found in the duodenum, 30% in the jejunum, and around 10% in the ileum.

The primary challenge in diagnosing these tumors is their gradual onset and nonspecific symptoms. Patients frequently present with vague abdominal discomfort and occasionally with signs of obstruction. Adenocarcinomas, in particular, may lead to cramp-like pain, similar to what our patient experienced, often due to partial obstruction. Among the 15 patients with data on their presenting symptoms, nine (60%) had vomiting, eight (53%) had abdominal pain, and three (20%) presented with weight loss, melena, and anemia, each. One patient (6%) reported postprandial fullness. Our patient presented with chief complaints of vomiting, abdominal pain, and melena, which are among the most common symptoms. Thus, features of intestinal obstruction, followed by gastrointestinal bleeding, are the common presentations in patients with DJ flexure tumors.

When comparing the radiological diagnostic modalities used, a mass was detected by CT in five patients (33.3%), with three of these (20%) showing associated intussusception. CT imaging revealed a thickened wall in two patients (12%), while stricturous lesions in the proximal jejunum and concentric luminal narrowing were found in one patient (6%). Barium meal and follow-through were used in two patients (12%), and ^68^Ga-DOTATATE uptake identified a mass in one patient (6%), each. In our case, a contrast-enhanced abdominal CT was done, which revealed edematous wall thickening in a segment of the proximal jejunal loop with adhesions. MR enterography confirmed similar findings and showed an abnormal communication between the D3 segment of the duodenum and the jejunal lumen, suggesting an enteroenteric fistula. This is not among the commonly reported findings. Thus, cross-sectional imaging will help localize the tumor site to plan surgical resection.

Of the 41 reported cases of DJ flexure tumors, gastrointestinal stromal tumor (GIST) was the most common diagnosis, found in 28 patients (68%). Among the 24 cases of GIST reporting tumor grades, 18 (75%) were low grade, and six (25%) were high grade. Adenocarcinoma was diagnosed in eight patients (19.5%), and there was one case (2%) each of leiomyosarcoma, leiomyoma, neuroendocrine carcinoma, mixed adenoneuroendocrine carcinoma, and pancreatic choristoma. Thus, low-grade GIST is the most common tumor located at the DJ flexure. Our patient was diagnosed with DJ flexure adenocarcinoma with a fistulous connection between the DJ flexure and the jejunum. Although adenocarcinoma is the second most common tumor after GIST, a fistulizing phenotype has not been described previously.

Regarding surgical interventions, resection and anastomosis were performed in 20 cases (50%), including five end-to-end anastomoses, two end-to-side anastomoses, and two side-to-side anastomoses. Eight patients (19.5%) underwent laparoscopic resection, while 11 patients (26.8%) underwent open resection. One patient (2.4%) each underwent pancreaticoduodenectomy with simultaneous resection and Whipple's procedure. In our case, the patient underwent surgical excision of the DJ flexure with the proximal jejunum and descending colon, along with a D3-to-jejunum anastomosis, Hartmann's procedure, a feeding jejunostomy (FJ), and an end descending colostomy. Thus, the choice of surgery in DJ flexure mass depends on the tumor's location, extent, and nature.

Concerning associated diseases with DJ flexure tumors, two cases of adenocarcinomas were reported to have long histories of celiac disease. Long-standing celiac disease predisposes to enteropathy-associated T-cell lymphoma and adenocarcinoma of the small intestine [23]. A previous population-based study from Sweden reported a 10-fold increased risk of adenocarcinoma of the small intestine in patients with celiac disease [24]. Other associated conditions with DJ flexure tumors included Peutz-Jeghers syndrome in one case of adenocarcinoma and neuroendocrine carcinoma and gastric GIST in a patient with DJ flexure GIST. Patients with Peutz-Jeghers syndrome have an increased risk of gastrointestinal malignancies, with the colorectal being the most common site, followed by the small intestine [25]. Thus, patients with DJ flexure tumors should be evaluated for associated conditions and actively managed for them.

Data concerning long-term outcomes of patients with DJ flexure tumors are limited. Among patients with adenocarcinomas, only four patients were followed up for durations varying from three months to 11 years, and one patient developed recurrence after five years, resulting in mortality. Among the 22 cases of GIST, only one recurrence was reported. Another two cases of poorly differentiated neuroendocrine carcinoma and leiomyosarcoma developed disease recurrence after four months and three years of resection, respectively. Thus, these patients should be placed on long-term clinical and radiological surveillance per standard guidelines to diagnose and treat early recurrence [26,27].

## Conclusions

To conclude, the DJ flexure is an uncommon location for small intestinal tumors, with only a few reported cases. DJ flexure tumors are associated with a myriad of symptoms, but intestinal obstruction and gastrointestinal bleeding are common symptoms. These tumors are usually visualized as mass or thickening on cross-sectional imaging, but there are no reports of enteroenteric fistula associated with DJ flexure tumor, making this case unique. Surgical resection remains the only curative option. Concomitant conditions predisposing to small intestinal tumors should be searched in patients with DJ flexure tumors. Further data are required on the long-term outcomes of these patients.
